# Cortisol and Phosphate Homeostasis: Cushing’s Syndrome Is Associated With Reversible Hypophosphatemia

**DOI:** 10.3389/fendo.2021.733793

**Published:** 2021-09-30

**Authors:** Ariadne Bosman, Annewieke W. van den Beld, Richard A. Feelders, M. Carola Zillikens

**Affiliations:** ^1^ Department of Internal Medicine, Erasmus University Medical Center, Rotterdam, Netherlands; ^2^ Department of Internal Medicine, Groene Hart Ziekenhuis, Gouda, Netherlands

**Keywords:** Cushing’s syndrome, cortisol, hypercortisolism, phosphate, hypophosphatemia, glucocorticoids

## Abstract

**Objectives:**

The influence of hypercortisolism on phosphate homeostasis is relatively unknown. A few previous studies have reported on patients with Cushing’s syndrome (CS) with hypophosphatemia in whom serum phosphate normalized after initiation of treatment for CS. We aimed to investigate the prevalence of hypophosphatemia in CS, the association between the degree of hypercortisolism and serum phosphate and the change in serum phosphate after remission of CS. We compared the prevalence of hypophosphatemia in CS with the prevalence in the population-based Rotterdam Study (RS).

**Methods:**

Patients diagnosed with CS and treated at the Department of Endocrinology of Erasmus MC in the period of 2002-2020 were included and data was collected on age at diagnosis, sex, serum phosphate, calcium and potassium levels, kidney function and BMI. Using multivariate linear regression, we analyzed the association between 24h urinary free cortisol excretion (UFC) and serum phosphate. Changes in serum phosphate and covariates were tested with a repeated measurement ANOVA, using mean levels of laboratory values for the periods before remission, and 0-14 days and 15-180 days after remission.

**Results:**

Hypophosphatemia before treatment was present in 16% of the 99 CS patients with data on serum phosphate, 24h UFC and covariates. In comparison, the prevalence of hypophosphatemia in RS was 2.0-4.2%. Linear regression showed a negative association between the level of UFC and serum phosphate at diagnosis, which remained significant after adjusting for covariates [β -0.002 (95%CI -0.004; -0.0004), p=0.021]. A subset of 24 patients had additional phosphate measurements at 0-14 days and 15-180 days after remission. In this subgroup, serum phosphate significantly increased from 1.03 ± 0.17 mmol/L prior to remission to 1.22 ± 0.25 mmol/L 15-180 days after remission (*p* = 0.008). BMI decreased after remission [-1.1 kg/m^2^, (95%CI -2.09 to -0.07), p=0.037]. Other covariates did not show an equivalent change over time.

**Conclusion:**

In this retrospective study, we found that 16% of patients with CS had hypophosphatemia. Moreover, serum phosphate was related to the level of cortisoluria and increased after remission of CS. Potential underlying mechanisms related to urinary phosphate excretion and possibly involving FGF23, BMI and parathyroid hormone levels should be further explored.

## Introduction

Cushing’s syndrome (CS) results from chronic exposure to either endogenous or exogenous excess of cortisol (1). A well-known complication of hypercortisolism in CS is glucocorticoid-induced osteoporosis (GOP) (2, 3). GOP is thought to be the result of a combination of decreased intestinal calcium absorption and renal calcium resorption, increased bone resorption, decreased bone formation and muscle wasting. Consequently, biochemical remission of Cushing’s syndrome results in an increase in bone mineral density (3). Recently, it has been suggested that treatment with glucocorticoids could also affect phosphate homeostasis and even induce hypophosphatemia due to increased urinary phosphate excretion (4, 5). Among drugs that are associated with hypophosphatemia, glucocorticoids have been suggested to be among the most common pharmacological agents associated with profound hypophosphatemia in hospitalized patients (6). Similarly, some case reports have described hypophosphatemia in patients with CS (7–9). After treatment for CS, normalization of serum phosphate levels has been reported after two weeks and can take up to one year (7–9). Findling et al. reported seven patients with CS who went in remission after treatment. One year after remission, they reported a significant increase in tubular reabsorption of phosphate, a reduction in daily urinary calcium excretion, a decrease in immunoreactive parathyroid hormone (PTH) and a decrease in 1,25 dihydroxy vitamin D (1,25(OH)_2_D) (9). Similar to glucocorticoid use, it has been hypothesized that hypercortisolism in CS can induce hypophosphatemia by increasing urinary phosphate excretion or by inhibiting intestinal phosphate absorption. This process may be mediated by Fibroblast Growth Factor 23 (FGF23) (8, 10, 11). Indeed, Delucchi et al. reported an association between sustained glucocorticoid treatment and increased intact FGF23 levels in pediatric renal transplant patients (12).

Phosphate is important for energy metabolism, cell signaling and oxygen transport. It is also a component of DNA and RNA, and it is critical for skeletal development and bone mineralization (13, 14). Most of phosphate in the human body is stored in bone, the remainder is localized in soft tissue (15). Phosphate deficiency can cause a variety of clinical problems such as muscle weakness, rickets in children and osteomalacia in adults (16). Phosphate homeostasis is regulated by several factors of which PTH, 1,25 dihydroxy vitamin D and FGF23 appear to be the most important (15, 17). Whereas knowledge of the role of phosphate and phosphate homeostasis is increasing, little is known about the relation between cortisol, and specifically Cushing’s syndrome (CS) and phosphate homeostasis.

The prevalence of hypophosphatemia in CS is currently unknown and the changes in phosphate concentrations after treatment for CS have only been studied in very small patient groups. Moreover, the role of potential confounders of phosphate homeostasis, such as BMI and kidney function, have not been adequately explored yet. In this study, we aim to evaluate the prevalence of hypophosphatemia in CS, the association between the level of 24h urinary free cortisol excretion (UFC), as a marker of the degree of hypercortisolism, and serum phosphate concentrations; the role of potential confounders and the change in serum phosphate levels after remission of CS. We compare the prevalence of hypophosphatemia in CS to the prevalence in a population-based cohort study of males and females.

## Materials and Methods

### Patients

This retrospective study included patients from the endocrinology department of the Erasmus University Medical Center, Rotterdam, the Netherlands, who were diagnosed with endogenous CS in the period 2002-2020. A diagnosis of CS was made based on three screening tests: late night salivary cortisol concentration, 24h UFC and the 1 mg overnight dexamethasone suppression test (1). In patients with adrenocorticotropin hormone (ACTH) dependent CS, a pituitary-dependent cause was differentiated from an ectopic cause by bilateral inferior petrosal sinus sampling in case of a non-visible adenoma on MRI or an adenoma less than 6 mm. In patients with ACTH-independent CS, CT or MRI was performed to image the adrenal glands. To study the prevalence of hypophosphatemia and the association between 24h UFC and serum phosphate concentration, we included patients for whom serum phosphate measurements were available that were taken after diagnosis and before remission. A total of 99 patients had complete data on serum phosphate, 24h UFC and covariates before remission and they were included to study the prevalence of hypophosphatemia and the association between UFC and serum phosphate. In addition, in the subset of this population in whom serum phosphate had also been measured after remission of CS, we studied the effect of treatment of CS on serum phosphate concentration. For 24 patients with serum phosphate measurements at time of diagnosis, serum phosphate measurements and covariates were available postoperatively and within 180 days after remission. Lastly, we determined the difference in serum phosphate concentration between the time of diagnosis and more than 180 days after remission. For this analysis we included 45 patients with a serum phosphate measurement that was taken at time of diagnosis and a serum phosphate measurement taken more than 180 days, but less than 3 years, after remission, when the patient either used hydrocortisone at a physiological dosage or supplementation had stopped. We repeated this analysis in 30 patients with additional information on covariates.

We compared the prevalence of hypophosphatemia in CS to the prevalence in the Rotterdam Study (RS). RS is a population-based study of males and females aged 40 or more from the district Ommoord in Rotterdam, the Netherlands. The rationale and design have been described in more detail elsewhere (18). This study is ongoing since 1990 and is now composed of four cohorts, named RS-I, RS-II, RS-III and RS-IV (initiated in 1990, 2000, 2005 and 2017). The Rotterdam Study has been approved by the Medical Ethics Committee of the Erasmus MC (registration number MEC 02.1015) and by the Dutch Ministry of Health, Welfare and Sport (Population Screening Act WBO, license number 1071272-159521-PG). All participants provided written informed consent to participate in the study and to have their information obtained from treating physicians. A total of 5,182 participants from RS-I, 2,511 from RS-II and 3,435 from RS-III with information on serum phosphate concentration were included to study the prevalence of hypophosphatemia in RS.

### Methods

Serum samples from patients were analyzed as part of standard care for CS, at the department of clinical chemistry of Erasmus MC. Prior to 2013, 24h UFC was measured with a chemiluminescence immunoassay using unextracted urine (Immulite XPi, Siemens AG, Munich, Germany). The upper limit of normal (ULN) of this assay was 850 nmol/24h. From 2013 onwards, UFC was measured using liquid chromatography/tandem mass spectrometry (LC/MSMS, Waters Xevo-TQ-S, Milford, MA). The ULN of this assay is 133 nmol/24h. Hypercortisolism was defined as cortisoluria higher than the ULN of 24 hour UFC. For the purpose of harmonisation for this study, the level of cortisoluria was defined as the times of ULN (xULN) of 24 hour UFC. Data on age, sex, cause of CS, level of cortisoluria at time of diagnosis, serum phosphate and Cushing related treatment were collected from the medical files. Furthermore, we collected data on serum creatinine, total calcium, potassium, body mass index (BMI), proton-pump inhibitors (PPI) use, thiazide and loop diuretics use, as these variables have been associated with phosphate homeostasis. BMI (kg/m^2^) was estimated from weight and height measured at clinical presentation. To calculate the estimated glomerular filtration rate (eGFR), the Chronic Kidney Disease Epidemiology Collaboration (CKD-EPI) equation was applied (19). Hypophosphatemia was defined as a serum phosphate concentration below 0.80 mmol/L (normal range: 0.80-1.40 mmol/L).

In the subset of the population with serum phosphate measurements after remission of CS, the treatment modalities leading up to remission varied. In this population, the date of remission was defined as follows: the date of biadrenalectomy or adrenalectomy; the date of removal of the ACTH producing tumor; the date of the transsphenoidal hypophysectomy resulting in remission and the date when cortisoluria was less than ULN in 24 hour urine in medically or radiologically treated patients.

### Statistical Analysis

The associations between 24h UFC and serum phosphate were examined through linear regression models, with the serum phosphate measurement that was taken nearest to the date of diagnosis modeled as the dependent variable and xULN of 24 hour UFC modeled as the independent variable. Analyses were adjusted for serum potassium, eGFR, total calcium, BMI, smoking and use of loop diuretics, thiazide diuretics and PPIs.

To analyze the difference between mean serum phosphate before remission and several time periods after remission, we applied a repeated measures ANOVA. Measurements of serum phosphate are not part of standard care for CS (20). Therefore, it was expected that serum phosphate had been measured at different time points and there would be missing data. To study the change in serum phosphate postoperatively and after several months, mean serum phosphate levels were calculated for the periods before remission, 0-14 days after remission and 15-180 days after remission and a repeated measures ANOVA was performed. Normality was assessed using Shapiro-Wilk’s test. Analyses were repeated after exclusion of any outliers in the data. Sphericity was tested with Mauchly’s test of sphericity. In the models chosen for statistical analysis, it was not possible to adjust for covariates. Therefore, any change in total calcium, potassium, eGFR and BMI was studied by comparing the means before and after remission using the statistical approach as described above. Covariates that do not show a change after remission are considered to have little or no effect on any change in serum phosphate concentrations that may be observed.

To determine the change in serum phosphate concentration in patients with a serum phosphate measurement taken at the time of diagnosis and more than 180 days after remission, we applied a paired student T-test. For this analysis we included the serum phosphate measurement that was taken nearest to the date of diagnosis and the first serum phosphate measurement that was taken more than 180 days, but less than 3 years, after remission, when the patient either used hydrocortisone at a physiological dosage or supplementation had stopped. A hydrocortisone dosage of 10 milligram in the morning, 5 milligram in the afternoon and 5 milligram in the evening was classified as physiological. We chose a cut-off of 3 years because we consider this time period to be reasonably unaffected by change due to other factors such as ageing (21)

Lastly, as a sensitivity analysis, we tested differences in serum phosphate, cortisoluria, serum calcium, potassium, eGFR, BMI and diuretics and PPI use between patients with and without hypophosphatemia and between patients with ACTH producing pituitary adenomas and ectopic ACTH production using chi-square and Mann-Whitney U tests.

Results are presented as mean ± SD, except where otherwise indicated. A p-value less than 0.05 was considered statistically significant. All analyses were performed with IBM SPSS software, version 25 (SPSS, Chicago, IL) and R version 3.6.1 (Vienna, Austria). The medical ethical committee of the Erasmus MC approved this study.

## Results

The general characteristics of the study population (N=99) and of the subset of the population with serum phosphate measurements at 0-14 days and 15-180 days after remission (N=24) are depicted in **Table 1**. In the total population, 73.7% was female and the mean ± SD age at diagnosis was 46.4 ± 13.5 years. An ACTH producing pituitary adenoma was diagnosed in 74.7% of patients, ectopic ACTH production was diagnosed in 23.2% of patients and 2.0% had adrenal CS. In the subset of the population with measurements at 0-14 days and 15-180 days after remission (N=24), 67% was female and the mean age at diagnosis was 50.3 ± 12.8 years. Of these 24 patients, 62.5% was diagnosed with an ACTH producing pituitary adenoma and 37.5% was diagnosed with ectopic ACTH production.

**Table 1:** General characteristics of the study population at time of diagnosis.

	All	With 0-14 and 15-180 day measurements
**N**	99	24
**Age at diagnosis, years**	46.4 (13.5)	50.3 (12.8)
**Female (%)**	73 (73.7%)	16 (67%)
**Phosphate, mmol/L**	1.01 (0.21)	1.04 (0.19)
**Hypophosphatemia (%)**	16 (16.2%)	2 (8.3%)
**Cortisoluria, xULN UFC median (min, max)**	2.6 (0.5, 144.3)	3.9 (0.6, 89.7)
**Calcium, mmol/L**	2.31 (0.21)	2.27 (0.18)
**Potassium, mmol/L**	4.0 (0.6)	3.9 (0.7)
**eGFR, mL/min/1.73m^2^**	97.7 (20.1)	100.6 (18.6)
**BMI, kg/m^2^**	29.0 (6.7)	28.9 (7.5)
**Thiazide diuretics use (%)**	20 (20.2%)	4 (16.7%)
**Loop diuretics use (%)**	4 (4.0%)	3 (12.5%)
**PPI use (%)**	24 (23.3%)	5 (20.8%)
**Current smoking (%)**	22 (21.4%)	5 (20.8%)
**Cause of hypercortisolism**		
**ACTH producing pituitary adenoma (%)**	74 (74.7%)	15 (62.5%)
**Ectopic ACTH production (%)**	23 (23.2%)	9 (37.5%)
**Adrenal CS (%)**	2 (2.0%)	–
**Treatment**		
**No remission (%)**	8 (8.1%)	–
**Hypofysectomy(%)**	22 (22.2%)	4 (16.7%)
**Medication(%)**	28 (28.3%)	5 (20.8%)
**Bilateral adrenalectomy(%)**	28 (28.3%)	13 (54.2%)
**Adrenalectomy (%)**	2 (2.0%)	–
**Carcinoid resection(%)**	4 (4.0%)	–
**Radiation therapy(%)**	6 (6.1%)	1 (4.2%)
**Unknown (%)**	1 (1.0%)	–

Results are presented as mean (standard error) for continuous variables and count (percentages) for categorical variables, unless otherwise stated. BMI, body mass index; CS, Cushing’s syndrome; eGFR, estimated glomerular filtration rate; PPI, protonpumpinhibitors; xULN UFC, the times of upper limit of normal of 24 hour urinary free cortisol.

### Prevalence of Hypophosphatemia

In the total population of CS patients, mean serum phosphate at time of diagnosis was 1.01 mmol/L ± 0.21. 16% of these patients had hypophosphatemia. In RS-I of the Rotterdam Study (n=5,182), 61.4% was female, mean ± SD age was 70.3 ± 9.0, mean serum phosphate level was 1.19 mmol/L ± 0.20 and hypophosphatemia was present in 2.0% of the population. In RS-II (n=2,511), 54.6% was female, mean ± SD age was 64.7 ± 7.8, mean serum phosphate level was 1.08 mmol/L ± 0.16 and hypophosphatemia was present in 4.2% of the population. In RS-III (n=3,435), 56.4% of patients was female, mean ± SD age was 57.1 ± 6.8, mean serum phosphate level was 1.12 mmol/L ± 0.17 and hypophosphatemia was present in 2.9% of the population.

### Association Between the Level of Cortisoluria and Serum Phosphate at Time of Diagnosis

Linear regression analyses showed a significant inverse association between serum phosphate at time of diagnosis and xULN of 24h UFC [β (95% CI): β= -0.003 (-0.005 to -0.002), p<0.001], which remained significant after adjustment for serum total calcium, potassium, eGFR, BMI, smoking, use of loop diuretics, thiazide diuretics and PPIs [β (95% CI): β= -0.002 (-0.004 to -0.0004), p=0.021]. Additional adjustment for age and sex did not change results (data not shown).

### Change in Serum Phosphate After Remission

**Figure 1:**
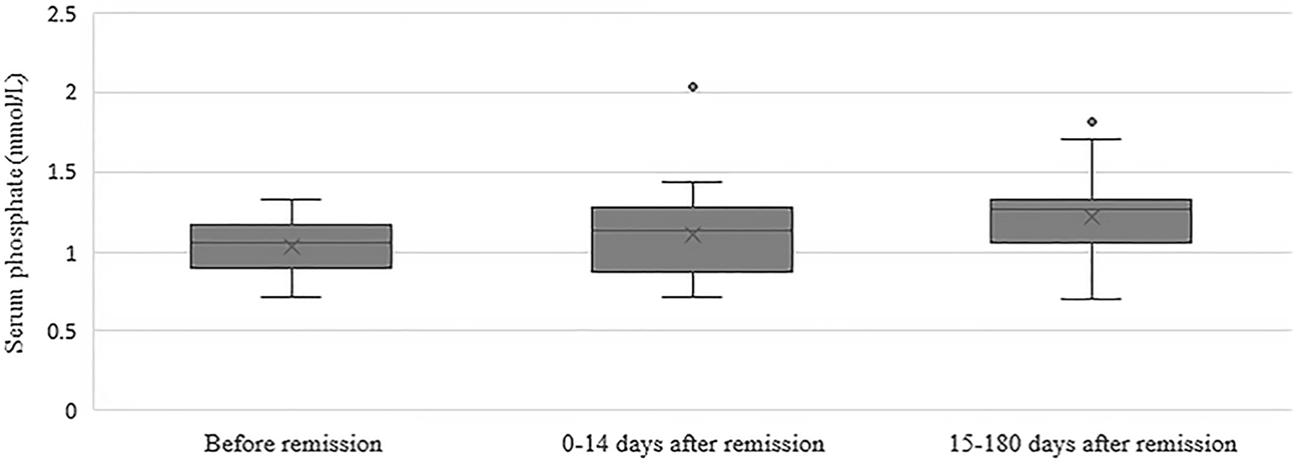
Box and whisker plots illustrating serum phosphate concentrations before remission, 0-14 days after remission and 15-180 days after remission. Boxes include medians and interquartile range. Whiskers extend 1.5 times the interquartile range.

**Figure 2:**
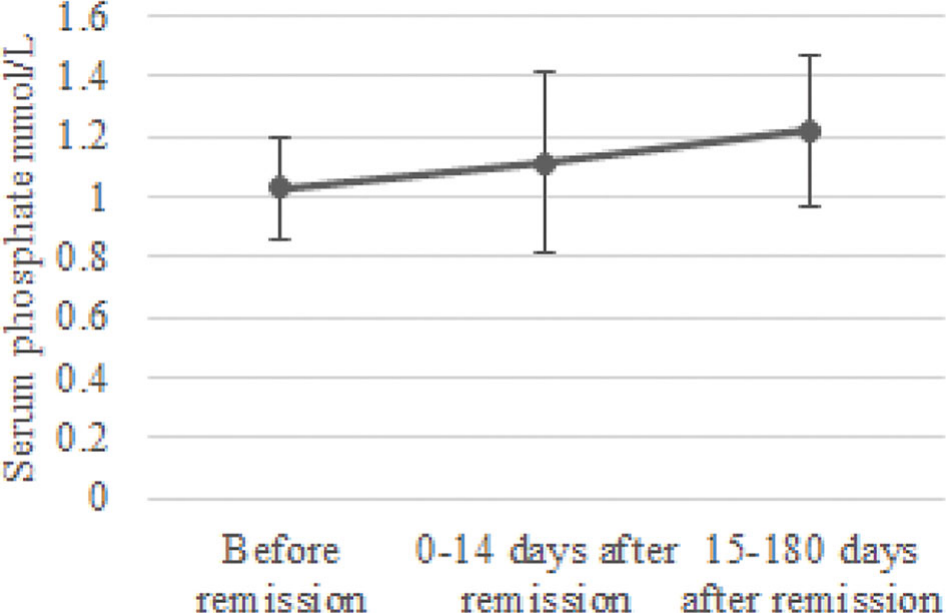
Change in serum phosphate concentration after remission. Mean serum phosphate levels and standard deviation were calculated for the periods before remission, 0-14 days after remission and 15-180 days after remission.

In the group of 24 patients with serum phosphate measurements after remission, mean serum phosphate was 1.03 ± 0.17 before remission, 1.11 ± 0.30 mmol/L at 0-14 days and 1.22 ± 0.25 mmol/L at 15-180 days after remission **Figure 1** depicts the box and whisker plots with the medians and interquartile range for the different time points. In this group, 8% had hypophosphatemia at time of diagnosis. A repeated-measures ANOVA showed that the mean phosphate levels were statistically significantly different between the different time points before and after remission F(2, 46) = 4,765, p = 0.013. A *post hoc* test using Bonferroni correction showed a substantial increase in serum phosphate from 1.03 mmol/L prior to remission to 1.22 mmol/L 180 days after remission, a significant increase of 0.19 (95%CI 0.04 to 0.33) mmol/L, p = 0.008 (**Figure 2**). Analysis was repeated after exclusion of outliers of serum phosphate and yielded similar results.

Next, we determined the difference in serum phosphate concentration between the time of diagnosis and more than 180 days after remission. In this group of 45 patients, serum phosphate increased significantly from 1.02 ± 0.18 mmol/L at time of diagnosis to 1.12 ± 0.25 mmol/L at >180 days after remission, a significant increase of 0.09 mmol/L (95%CI 0.02 to 0.17, p=0.019). Results were similar when restricting the analysis to patients who also had eGFR, serum total calcium, potassium and BMI measured more than 180 days after remission: increase of 0.11 mmol/L (95%CI 0.001 to 0.21), p=0.051, N=30).

### Changes in Covariates After Remission

**Figure 3:**
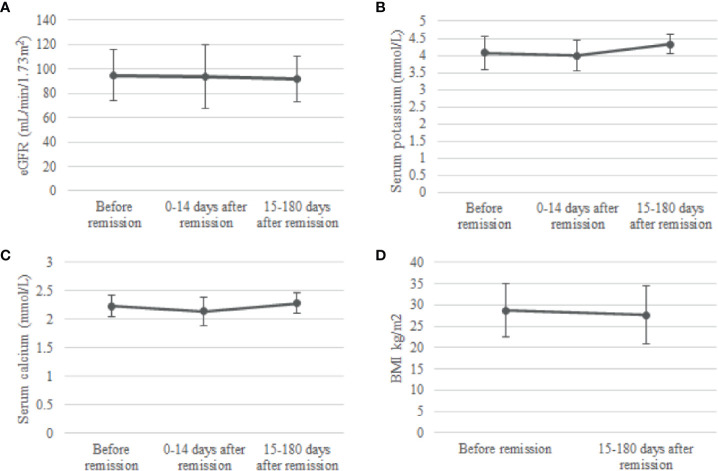
Change in eGFR **(A)**, serum potassium **(B)**, serum calcium **(C)** and BMI **(D)** after remission. Means and standard deviations were calculated for the periods before remission, 0-14 days after remission and 15-180 days after remission. eGFR, estimated glomerular filtration rate.

In the group op 24 patients with phosphate measurements 0-14 days and 15-180 days after remission, no change was observed in eGFR after remission of CS. There was a slight increase in serum potassium concentration from 3.99 ± 0.45 mmol/L 0-14 days after remission to 4.33 ± 0.28 mmol/L 15-180 days after remission, a significant increase of 0.34 (95%CI, 0.07 to 0.61) mmol/L, p = 0.009. Moreover, serum total calcium increased from 2.14 ± 0.25 mmol/L at 0-14 days after remission (p=0.046) to 2.28 ± 0.18 mmol/L at 15-180 days after remission (p=0.034). Lastly, we observed a significant decrease in BMI when comparing BMI at 15-180 days after remission with BMI before remission [paired t-test: -1.1 (95%CI -2.09 to -0.07), p=0.037] (**Figure 3**).

In the group of 30 patients with serum phosphate, total calcium, potassium, eGFR and BMI measurements taken at time of diagnosis and more than 180 days after remission, no change was observed in serum calcium. Interestingly, eGFR decreased from 98.83 ± 19.66 mL/min/1.73m^2^ to 83.30 ± 24.39 mL/min/1.73m^2^, a significant decrease of 15.53 mL/min/1.73m^2^ (95%CI 8.07 to 22.97, p-value <0.001), while serum potassium increased from 4.11 ± 0.57 mmol/L to 4.46 ± 0.38 mmol/L, a significant increase of 0.35 (95%CI 0.05 to 0.64, p-value=0.022). BMI decreased from 30.2 ± 1.4 to 28.2 ± 1.5, a significant decrease of 2.0 (95%CI -3.0 to -0.9, p-value=0.001).

Lastly, differences between patients with hypophosphatemia and without hypophosphatemia and between patients with ACTH producing pituitary adenomas and ectopic ACTH production were tested using chi-square and Mann-Whitney U tests. Differences between patients with hypophosphatemia and without hypophosphatemia are depicted in **Table 2**. Here, xULN of 24h UFC was higher in patients with hypophosphatemia than in patients without hypophosphatemia (p=0.024). Patients with hypophosphatemia had lower serum calcium levels (p<0.001) and were more likely to have CS from ectopic ACTH production than patients without hypophosphatemia. Differences between patients with ACTH producing pituitary adenomas and with ectopic ACTH production are depicted in **Table 3**. Patients with CS from ectopic ACTH production were older (p=0.031), had lower phosphate concentration at time of diagnosis (p=0.004), were more likely to have hypophosphatemia (p=0.023), had higher xULN of 24h UFC (p<0.001) and had lower potassium concentrations (p<0.001) than patients with CS from ACTH producing pituitary adenomas.

**Table 2:** Differences between patients with hypophosphatemia and with normal phosphate concentration before remission.

	Hypophosphatemia	Normal phosphate	*P*
**N**	16	83	
**Age at diagnosis in years**	49.9 (19.7)	46.5 (19.5)	0.849
**Female (%)**	13 (81.3%)	60 (72.3%)	0.550
**Phosphate, mmol/L**	0.68 (0.13)	1.06 (0.17)	<0.001
**Cortisoluria, xULN UFC**	5.6 (53.9)	2.6 (3.0)	0.022
**Calcium, mmol/L**	2.17 (0.37)	2.33 (0.21)	0.003
**Potassium, mmol/L**	4.0 (0.9)	4.1 (0.7)	0.362
**eGFR, mL/min/1.73m^2^**	101.7 (29.5)	100.1 (26.6)	0.680
**BMI, kg/m^2^**	26.8 (6.6)	27.6 (9.4)	0.356
**Thiazide diuretics use (%)**	0	20 (24.1%)	0.037
**Loop diuretics use (%)**	2 (12.5%)	2 (2.4%)	0.121
**PPI use (%)**	6 (37.5%)	17 (20.5%)	0.126
**Current smoking (%)**	3 (18.8%)	17 (20.5%)	0.590
**Cause of hypercortisolism**			
** ACTH producing pituitary adenoma (%)**	8 (50.0%)	66 (79.5%)	0.044
** Ectopic ACTH production (%)**	7 (43.8%)	16 (19.3%)	
** Adrenal CS (%)**	1 (6.3%)	1 (1.2%)	

Results are presented as median (interquartile range) for continuous variables and count (percentages) for categorical variables. ACTH, adrenocorticotropin hormone; BMI, body mass index; CS, Cushing’s syndrome; eGFR, estimated glomerular filtration rate; PPI, protonpumpinhibitors; xULN UFC, the times of upper limit of normal of 24 hour urinary free cortisol.

**Table 3:** Differences between patients with an ACTH producing pituitary adenoma and ectopic ACTH production before remission.

	ACTH producing pituitary adenoma	Ectopic ACTH production	*P*
**N**	74	23	
**Age at diagnosis in years**	44.6 (17.7)	54.6 (22.6)	0.031
**Female (%)**	55 (74.3%)	16 (69.6%)	0.788
**Phosphate, mmol/L**	1.06 (0.21)	0.92 (0.30)	0.004
**Hypophosphatemia**	8 (10.8%)	7 (30.4%)	0.042
**Cortisoluria, xULN UFC**	2.3 (2.1)	19.1 (42.1)	<0.001
**Calcium, mmol/L**	2.35 (0.20)	2.22 (0.24)	0.005
**Potassium, mmol/L**	4.1 (0.6)	3.7 (1.1)	<0.001
**eGFR, mL/min/1.73m^2^**	99.4 (27.9)	106.9 (20.9)	0.031
**BMI, kg/m^2^**	28.2 (9.0)	24.5 (4.7)	0.004
**Thiazide diuretics use (%)**	20 (27.0%)	0	0.003
**Loop diuretics use (%)**	3 (4.1%)	1 (4.3%)	1.0
**PPI use (%)**	16 (21.6%)	7 (30.4%)	0.408
**Current smoking (%)**	17 (23.0%)	3 (13.0%)	0.387

Results are presented as median (interquartile range) for continuous variables and count (percentages) for categorical variables. ACTH, adrenocorticotropin hormone; BMI, body mass index; eGFR, estimated glomerular filtration rate; PPI, protonpumpinhibitors; xULN UFC, the times of upper limit of normal of 24 hour urinary free cortisol.

## Discussion

In this study we investigated the prevalence of hypophosphatemia in CS, and the change in serum phosphate concentration and potential confounders of phosphate homeostasis after remission of CS. In addition, we explored the association between 24h UFC and serum phosphate before remission. Data from our study showed that hypophosphatemia was present in up to 16% of our patients with active CS. The prevalence of hypophosphatemia in these patients is four to six times higher than in participants from a population-based cohort study. We also found that serum phosphate increases after remission, which also suggests that hypercortisolism affects serum phosphate concentration. The level of cortisoluria found in hypophosphatemic patients and the inverse association between the 24h UFC level and serum phosphate concentration indicate modulatory effects of cortisol on phosphate homeostasis.

Our results indicate that hypercortisolism in CS affects serum phosphate even to the extent of causing hypophosphatemia. Hypophosphatemia can cause multiple symptoms such as fatigue and muscle weakness, which are complaints that are commonly reported by CS patients. Nearly 60% of patients with Cushing’s syndrome have muscle weakness (22). Glucocorticoid induced myopathy is caused by an altered protein metabolism, resulting in muscle atrophy and muscle protein catabolism (22). In addition, it has been suggested that hypophosphatemia causes a decrease in muscle ATP synthesis (23). Consequently, hypophosphatemia may worsen muscle weakness in patients with CS and may also contribute to the development of glucocorticoid-induced low bone mineral density and fractures by causing osteomalacia. As can be expected, patients with CS based on ectopic ACTH production had higher 24h UFC levels than patients with CS due to ACTH producing pituitary adenomas (24), and were in turn more likely to develop hypophosphatemia.

Our findings are in line with and extend previous reported cases of hypophosphatemia in CS, in whom remission of CS resulted in normalization of serum phosphate (7, 8). Similarly, Findling et al. reported previously an increase in serum phosphate concentration after treatment of ACTH-dependent CS in 7 patients. However, the pathophysiological mechanism(s) for these changes in serum phosphate concentrations is largely unknown. Previous studies have suggested that glucocorticoids may reduce intestinal absorption of phosphate and increase renal phosphate excretion (4, 10). Indeed, Findling et al. observed an increase in the tubular reabsorption rate of phosphate (TRP) after treatment for CS.

**Figure 4:**
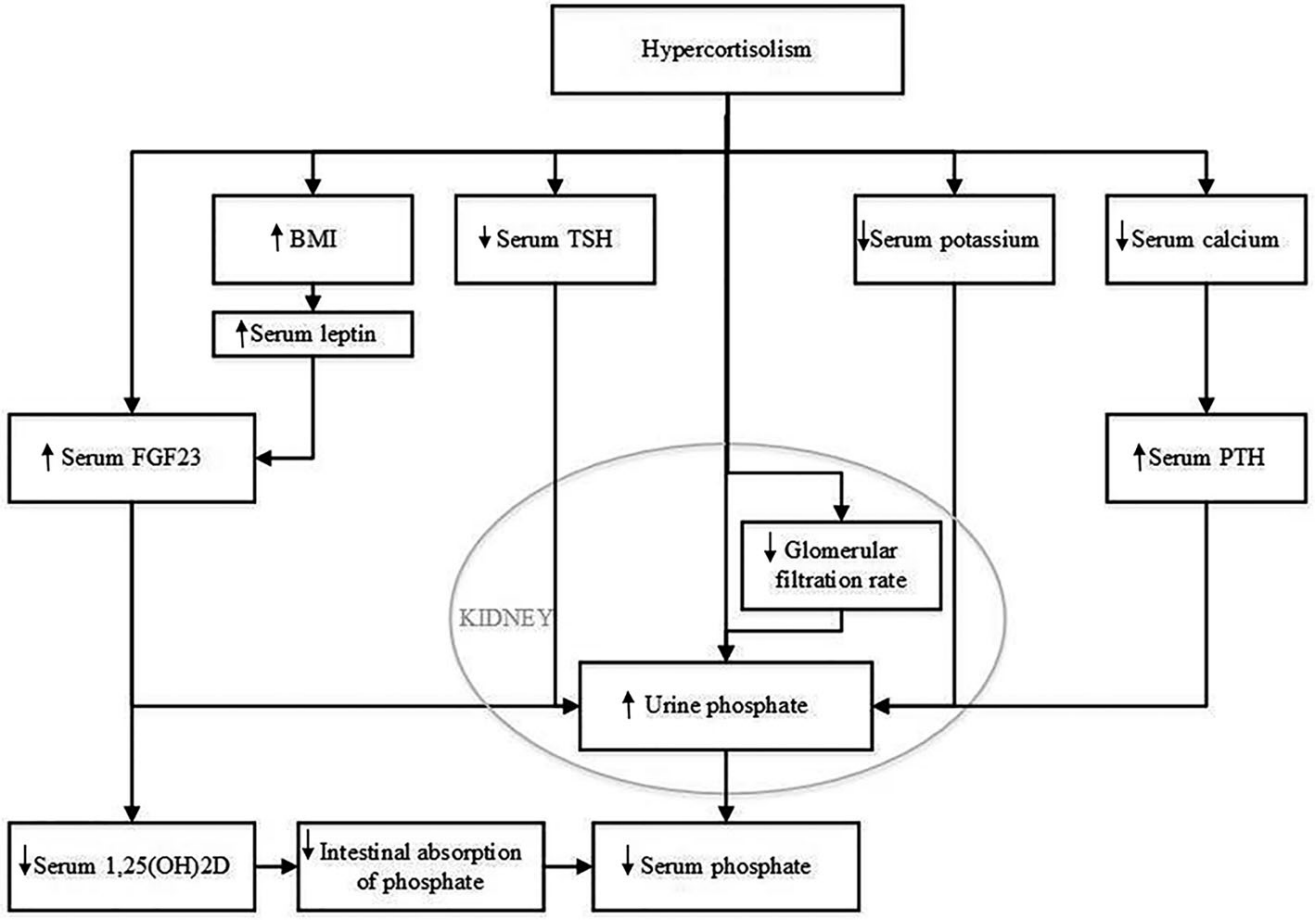
Potential mechanisms that could explain the effect of hypercortisolism on serum phosphate concentration. BMI, body mass index; FGF23, Fibroblast Growth Factor 23; PTH, parathyroid hormone; TSH, thyroid-stimulating hormone; 1,25(OH)2D, 1,25 dihydroxy vitamin D.

There are several potential hypothetical mechanisms that could explain the effect of glucocorticoids on serum phosphate concentration. These are summarized in **Figure 4**. One pathophysiological mechanism relates to FGF23, which is mainly expressed and secreted by osteocytes and osteoblasts (8, 10, 11). Expression of FGF23 is regulated by serum phosphate concentration. FGF23 regulates serum phosphate by e.g., increasing urinary phosphate excretion, but the role of glucocorticoids in FGF23 regulation is still unclear. Delucchi et al. reported an association between sustained glucocorticoid treatment and increased intact FGF23 levels in pediatric renal transplant patients (12). The same group reported an increase in bone FGF23 protein abundance and in FGF23 expression in MG53 cells, a human osteosarcoma cell line, when incubated with dexamethasone (12). In contrast, Feger et al. reported a downregulation of FGF23 transcription and protein synthesis in UMR106 rat osteoblast-like cells and MC3T3-E1 cells after incubation with dexamethasone or prednisolone. Similarly, injection of dexamethasone or prednisolone in mice lead to a decrease of serum C-terminal and intact FGF23 concentration and bone FGF23 mRNA expression, but, strikingly, also to increased renal phosphate excretion and decreased serum phosphate concentration, without affecting PTH (25). The authors state that their findings could be explained by the inhibitory effect of dexamethasone on membrane expression of sodium-dependent phosphate transporters in the kidney, resulting in increased renal phosphate excretion, as was previously reported (26). FGF23 is not routinely measured in patients with CS but Endo et al. reported a patient with hypophosphatemia due to ectopic ACTH production whose active FGF23 concentration was below the mean value previously found in healthy adults (11). We also recently observed normal C-terminal FGF23 levels in a patient who was diagnosed with hypophosphatemia and adrenal CS (unpublished observations). In this patient, serum phosphate concentration also recovered after adrenalectomy. These findings would support the hypothesis that the effect of glucocorticoids on serum phosphate concentration is independent of FGF23 and thus might be related to an effect of GCs on the sodium-dependent phosphate transporters. However there is clearly a need for larger studies on intact and C-terminal FGF23 before and after treatment of CS.

A second pathophysiological mechanism might relate to BMI. The majority of CS patients develop obesity (1, 27). Although the treatment of CS has been shown to lower BMI, patients treated for CS maintain a higher BMI than controls matched by sex and age (28, 29). Indeed, our study showed a decrease in BMI after treatment for CS. Previous literature has shown that BMI and serum phosphate levels are inversely associated (30, 31). Moreover, we recently observed evidence for a causal effect of BMI on serum phosphate using a Mendelian Randomisation approach (unpublished data). There are several theories on the pathophysiological mechanism behind this effect. A higher BMI is associated with lower 25-hydroxyvitamin D levels (32), which in turn could result in lower levels of 1,25(OH)_2_D leading to impaired phosphate absorption from the intestine. FGF23 may also play a role in adiposity associated decreases in serum phosphate as adiposity has also been associated with FGF23. Leptin, which has been shown to function as a FGF23 secretagogue, is strongly related to adiposity (33–35). Put differently, the change in serum phosphate levels after treatment for CS may be, at least in part, due to the decrease in BMI.

A third potential mechanism may involve kidney function. An important consequence of chronic hypercortisolism is the increased risk for cardiovascular complications, including atherosclerotic vascular damage (28). In a case-control study in 18 patients, Haentjes et al. showed that patients with Cushing’s disease have a decreased glomerular filtration rate compared to controls (36). Early stages of chronic kidney disease are associated with increased FGF23 levels and hyperphosphaturia (37). However, in these early stages of chronic kidney disease, serum phosphate levels are still maintained in the normal range. Hence, this would not explain why CS patients are more likely to develop hypophosphatemia. We did not observe a change in estimated glomerular filtration when we compared eGFR at time of diagnosis with 0-14 days and 15-180 days after remission. In contrast, we observed a decline in eGFR more than 180 days after remission.

A fourth possible pathophysiological mechanism that we considered involves serum potassium. Rat studies have shown that a potassium deficiency can result in phosphaturia (38). Similarly, in humans, potassium supplementation leads to a decrease in FGF23 and an increase in serum phosphate levels (39). Hypokalaemia can occur in any patient with CS (40). Due to hypercortisolism, the 11β-hydroxysteroid dehydrogenase type 2 enzyme, which converts cortisol into cortisone, can get saturated. Saturation of this enzyme results in activation of mineralocorticoid receptors, which results in increased renal excretion of potassium. Although we observed a slight increase in serum potassium concentration from 0-14 days after remission to 15-180 days after remission, we did not find a significant difference when comparing serum potassium before remission with serum potassium after remission.

A fifth potential pathophysiological mechanism involves serum calcium. Both serum calcium and serum phosphate levels are regulated by 1,25(OH)_2_D and PTH. It has been postulated that glucocorticoids inhibit calcium absorption from the intestinal tract, but this effect remains controversial (41). In the case series of Findling et al, serum calcium did not change, but there was a reduction observed in urinary calcium excretion after treatment for CS. Interestingly, we observed a decrease in serum calcium levels at 0-14 days after remission compared to before remission. This decrease however was not seen for the period of 15-180 days after remission. In theory it is still possible that increased urinary calcium excretion combined with decreased intestinal absorption during active CS results in secondary hyperparathyroidism with an increase in urinary P excretion. Unfortunately, serum PTH levels were not measured in our patients because serum calcium was normal.

Other hypothetical mechanisms that could be considered include the role of hypothalamic-pituitary axes such as the hypothalamic-pituitary-thyroid axis. Thyroid-stimulating hormone and thyroid hormone can be influenced by glucocorticoid excess which may affect serum phosphate homeostasis (41–43). Most of our patients had ACTH dependent Cushing’s syndrome. There is evidence that ACTH influences bone mass (44, 45), but a direct effect of ACTH on phosphate homeostasis remains to be elucidated.

This study has several limitations. A major limitation is the retrospective nature of the study and the considerable number of missing data. Because serum phosphate was not measured at set time points, we calculated mean serum phosphate levels. We can assume that this will negatively affect the variance of phosphate over time. To draw conclusions on the course of the phosphate levels over time we calculated several time points, including 0-14 days and 15-180 days after remission. It is not known at what time during the day the blood samples were drawn, which could affect serum phosphate levels (46). Finally, serum FGF23, 1,25(OH)_2_D, PTH nor urinary phosphate concentrations were available to us.

In conclusion, we showed that hypophosphatemia can occur in up to 16% of patients with CS, that serum phosphate concentration is related to the degree of hypercortisolism and that remission of CS results in an increase in serum phosphate. Effects were stronger in patients with CS due to ectopic ACTH production. These results suggest that hypercortisolism in CS affects phosphate homeostasis. We postulate that hypophosphatemia in CS patients may contribute to fatigue, muscle weakness and impaired bone quality. Therefore, the effect of hypercortisolism on FGF23 and urinary phosphate excretion should be further evaluated in a prospective setting and all patients with CS should be evaluated for hypophosphatemia, especially when it concerns CS from ectopic ACTH production.

## Data Availability Statement

The datasets presented in this article are not readily available because of restrictions based on privacy regulations. Requests to access the datasets should be directed to CZ, m.c.zillikens@erasmusmc.nl.

## Ethics Statement

The studies involving human participants were reviewed and approved by Medical Ethics Committee of the Erasmus MC. Written informed consent for participation was not required for the retrospective study in accordance with the national legislation and the institutional requirements. All participants from the Rotterdam Study provided written informed consent to participate in the study and to obtain information from their treating physicians.

## Author Contributions

AB, AvB, RF and CZ designed the research. AB conducted the research, performed the statistical analysis, and wrote the paper. RF provided the essential databases. AB and CZ have primary responsibility for final content. All authors contributed to the article and approved the submitted version.

## Funding

AB is supported by a grant from Health~Holland (PhosphoNorm, LSHM18029). The funders played no role in the study design or in data collection and analysis. The Rotterdam Study is funded by Erasmus Medical Center and Erasmus University, Rotterdam, Netherlands Organization for the Health Research and Development (ZonMw), the Research Institute for Diseases in the Elderly (RIDE), the Ministry of Education, Culture and Science, the Ministry for Health, Welfare and Sports, the European Commission (DG XII), and the Municipality of Rotterdam.

## Conflict of Interest

The authors declare that the research was conducted in the absence of any commercial or financial relationships that could be construed as a potential conflict of interest.

## Publisher’s Note

All claims expressed in this article are solely those of the authors and do not necessarily represent those of their affiliated organizations, or those of the publisher, the editors and the reviewers. Any product that may be evaluated in this article, or claim that may be made by its manufacturer, is not guaranteed or endorsed by the publisher.
